# Complete genome sequence of *Bacillus subtilis* strain S-LA1, a potential plant probiotic endophyte from the medicinal plant *Leucas aspera*

**DOI:** 10.1128/mra.00551-26

**Published:** 2026-08-17

**Authors:** Md. Shahrear Parvaj Sujon, Sharmin Islam, Avi Kumar Badhon, Jannatul Ferdous Eva, Dipali Rani Gupta, Tofazzal Islam

**Affiliations:** 1 Institute of Biotechnology and Genetic Engineering (IBGE), Gazipur Agricultural University (GAU) 198780, Gazipur, Bangladesh; DOE Joint Genome Institute, Berkeley, California, USA

**Keywords:** complete genome, Oxford Nanopore sequencing, endophytic bacteria, plant probiotic, *Bacillus*

## Abstract

*Bacillus subtilis* strain S-LA1 is an endophytic bacterium isolated from *Leucas aspera* roots that harbors a 4.2 Mbp genome predicted to encode several traits for nutrient acquisition, plant growth promotion, and plant probiotic efficacy. Genomic characterization underscores its potential as a microbial resource supporting sustainable agriculture and crop disease management strategies.

## ANNOUNCEMENT

*Bacillus subtilis* is a gram-positive bacterium widely distributed in soil, rhizosphere, and agricultural environments, colonizing plants as epiphytes or endophytes. It is well recognized for plant growth-promoting traits, such as phosphate solubilization, siderophore-production, nitrogen fixation, and phytohormone secretion (1, 2). It also produces antimicrobial metabolites, enabling biocontrol against fungal pathogens. Notably, strain BS45 antagonizes *Fusarium graminearum* (3), while KC14-1 shows broad antifungal activity against *Rhizoctonia solani* and *Valsa mali* (4). These traits highlight the species' agricultural importance.

We report the complete genome of *B. subtilis* strain S-LA1 isolated from surface-sterilized root tissues of *Leucas aspera* collected 23 May 2025 in Birol Upazila, Dinajpur, Bangladesh (25.63° N, 88.55° E). Two grams of roots was collected from multiple plants and surface-sterilized with 70% ethanol and 2% sodium hypochlorite, rinsed thrice in sterile distilled water, and then aseptically macerated. The homogenate was serially diluted to 10^−5^ and 100 µL was plated on nutrient agar (Liofilchem, Italy; Catalog-Number: 610036). Distinct colonies were purified by repeated streaking and incubated aerobically at 28°C for 48 h. A single colony was sub-cultured in nutrient broth (Liofilchem, Italy; Catalog-Number: 610037) and incubated at 28°C for 48 h at 180 rpm. Genomic DNA was extracted using the Tissue Genomic DNA Extraction HE Mini Kit (FAVORGEN, Taiwan) and quantified with a Qubit 4.0 fluorometer (Thermo Fisher Scientific). Sequencing libraries prepared with ONT kits (SQK-NBD114.24, Barcoding Kit 96 V14) were sequenced on a MinION R10.4.1 flow cell following the manufacturer’s protocol. No DNA shearing or size selection was performed. Base-calling was performed using Dorado v1.2.0 (SUP model).

Sequencing generated 29,353 reads totaling 212,191,429 bp raw reads (N_50_ 11,114). Reads were quality-checked with NanoPlot v1.46.1 (5) and adapters trimmed via Porechop v0.2.4 (6) and filtered (>1 kb) using NanoFilt v2.8.0 (7). *De novo* assembly was performed with Flye v2.9.5 (8) and polished with Racon v1.5.0 (9) and Medaka v2.0.1 (10). Genome quality was assessed with QUAST v5.2.0 (11) and completeness with CheckM v1.2.4 (12) using the *B. subtilis* CheckM marker set. Annotation was performed with the National Center for Biotechnology Information (NCBI) Prokaryotic Genome Annotation Pipeline v6.10 (13) and the RAST server v2.0 (14), while antiSMASH and BAGEL annotations were run independently on the assembled genome sequence. For all software, default parameters were used unless noted.

The *B. subtilis* strain S-LA1 comprises a 4.2 Mbp genome assembled as one contig, with 43.5% guanine-cytosine (GC) content. The NCBI annotation predicted 4,299 CDSs (total), including 4,189 protein-coding genes, and 86 tRNAs (Table 1). The NCBI average nucleotide identity (ANI) analysis confirmed closest similarity to *B. subtilis* (ANI 98.76%, assembly coverage 94.53%, type assembly coverage 93.78%; GCA_001697265.1) (15).

**TABLE 1:** Genomic features of *B. subtilis* strain S-LA1 isolated from the tissues of *L. aspera* roots

Genome feature	S-LA1
SRA	SRR37520458
GenBank accession	JBTKTN000000000
BioSample accession	SAMN54376224
16s accession	PX316690
GenBank assembly	GCF_055690685
Assembled genome size (bp)	4,177,895
GC content (%)	43.5
Coverage (×)	47.0×
Genome completeness (%)	98.15% (CheckM)
Contamination (%)	1.29%
N_50_ value (bp)	4,177,895
Number of contigs	1
Genes (total)	4,417
Coding sequences (CDSs)	4,299
CDSs (with protein)	4,189
CDSs (without protein)	110
Genes (RNA)	118
rRNAs	27 (9 × 5S, 9 × 16S, 9 × 23S)
Complete rRNAs	27 (9 × 5S, 9 × 16S, 9 × 23S)
tRNAs	86
ncRNAs	5
Pseudogenes (total)	110
Pseudogenes (ambiguous residues)	0
Pseudogenes (frameshifted)	69
Pseudogenes (incomplete)	52
Pseudogenes (internal stop)	18
Pseudogenes (multiple problems)	24
Number of subsystems (metabolic features)	332

BAGEL4 (16) detected antibacterial bacteriocin and RiPP clusters encoding Subtilosin_A, Subtilosin_(SboX), and Sublancin. AntiSMASH v8.0.4 (17) predicted 10 secondary metabolite gene clusters, including surfactin, bacillaene, fengycin, bacillibactin, and bacilysin. Predictive complete NRPS operons for surfactin (*srfAA-AD*) and plipastin (*ppsA*, *ppsE*, *ppsR*) highlight lipopeptide-mediated antifungal potential. Genes predicted to encode for cell wall-degrading enzymes (*csn*, *bglS*, *eglS*, *nprE*, *bpr*), exopolysaccharide biosynthesis (*eps* cluster), and phytase (*phy*) suggest root colonization and phosphorus mobilization (18). Collectively, these predictive genomic traits highlight strain S-LA1 as a potential candidate for biocontrol and plant growth promotion in sustainable agriculture.

## Data Availability

The whole-genome sequence of *Bacillus subtilis* strain S-LA1 has been deposited in NCBI GenBank (JBTKTN000000000), GenBank assembly accession (GCF_055690685), and the Sequence Read Archive (SRA) (SRR37520458) under BioProject (Mining Bangladesh Biogold) accession PRJNA1055760. Additional annotations generated using RAST, BAGEL, and antiSMASH have been deposited in Zenodo and are accessible at https://doi.org/10.5281/zenodo.18950844.
